# Portable, smartphone-assisted turn-on fluorescence sensor based on green-synthesized beetroot-derived carbon dots for alpha-cypermethrin monitoring in field samples

**DOI:** 10.1039/d5ra09623e

**Published:** 2026-04-22

**Authors:** Hersh J. Noori, Kamal M. Mahmood

**Affiliations:** a College of Science, Chemistry Department, University of Salahaddin Zanko St 44001 Erbil Iraq; b College of Science, Chemistry Department, University of Garmian Bardasor St 46021 Sulaimani Iraq Hersh.jalil@garmian.edu.krd

## Abstract

Conventional detection of α-cypermethrin (α-CYP), a widely used pyrethroid insecticide, often requires costly laboratory instrumentation, limiting its suitability for rapid, on-site monitoring. Here, we present, for the first time, a green, low-cost, and portable fluorescence sensing platform based on carbon dots (CDs) synthesized from red beetroot *via* a microwave digestion system. The CDs exhibited a strong green emission (*λ*_ex 347 nm, *λ*_em 450 nm) and displayed a unique turn-on fluorescence response to α-CYP, attributed to hydrophobic interactions and aggregation-induced emission enhancement (AIEE), as confirmed by solvent-dependent fluorescence studies, concentration studies, and TEM analysis. Under optimized pH and reaction conditions, the probe achieved a limit of detection (LOD) of 0.55 µmol L^−1^ and a limit of quantitation (LOQ) of 1.68 µmol L^−1^. The method demonstrated high selectivity against other common pesticides, with recoveries of 92–98% and relative standard deviations (RSDs) below 3.5% in spiked orange samples. Its integration with a smartphone RGB analysis application enabled field-deployable quantification, achieving an LOD of 0.71 µmol L^−1^ for on-site measurements. This work introduces a sustainable sensing strategy that combines green nanomaterials, smartphone-based analysis, and field validation, offering a practical solution for rapid pesticide detection in food safety and environmental monitoring.

## Abstract 2

Conventional detection of α-cypermethrin (α-CYP), a widely used pyrethroid insecticide, often requires costly laboratory instrumentation, limiting its suitability for rapid, on-site monitoring.

## Graphical abstract

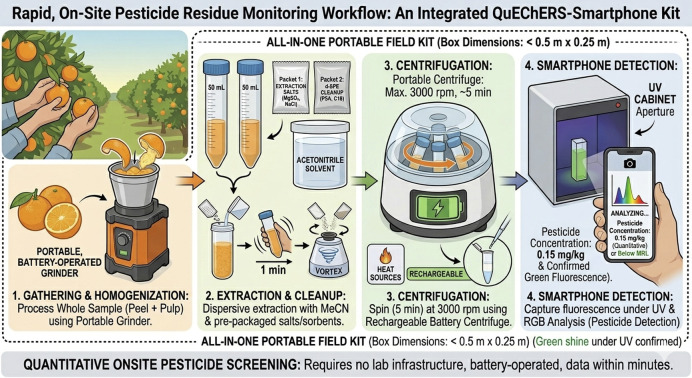

## 1 Introduction

**Fig. 1:**
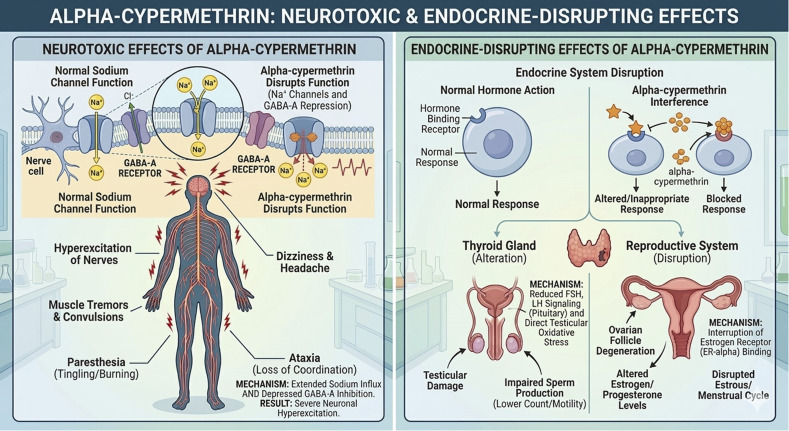
α-CYP's neurotoxic and endocrine effects.

In agriculture, pesticides are frequently used to protect crops from diseases and pests, resulting in increased yields and higher-quality food. Among these, α-cypermethrin (α-CYP), a type-II pyrethroid insecticide, is widely used to protect crops, such as cotton, rice, fruits, and vegetables.^1^ Despite its high efficacy and broad-spectrum activity, α-CYP is toxic to both terrestrial and aquatic organisms, bioaccumulative, and persistent in the environment. It has been linked to neurotoxic effects and possible endocrine disruption in humans (Fig. 1). For the sake of ecological safety and public health, the precise monitoring of α-CYP residues in food and environmental samples is therefore crucial.^2^ It is also rather persistent and bioaccumulative. Moreover, cypermethrin is known as an endocrine disruptor; for instance, α-CYP and its isomers have been shown to antagonize steroid and thyroid hormone receptors *in vitro*.^3^ These combined neurotoxic and endocrine-disrupting effects in humans, animals, and even plants highlight the need for the consistent monitoring of α-CYP residues in food and environmental samples.^4^

Chromatographic methods, including gas chromatography (GC),^5^ high-performance liquid chromatography (HPLC),^6,7^ and liquid chromatography-mass spectrometry (LC-MS),^6^ are the main techniques currently used for α-CYP detection. Despite their high sensitivity and specificity, these techniques are typically not appropriate for quick, on-site monitoring and call for expensive equipment, skilled workers, and substantial sample preparation. To overcome some of these restrictions, various sensing techniques have been developed, such as spectrofluorometric methods,^8,9^ flow injection analysis-photo-induced fluorescence (FIA-PIF),^10^ chemiluminescence,^11^ and molecularly imprinted polymers (MIPs).^12–14^ Unfortunately, their field applicability is limited because they frequently require laboratory-based analysis, multi-step fabrication, or synthetic reagents.

Recent developments in nanomaterials, which provide chemical stability, water solubility, and adjustable optical characteristics, have made it possible to use carbon dots (CDs) and quantum dots in fluorescence-based detection systems. Their potential for in-field pesticide detection has been shown by the integration of some of these systems with smartphones for portable analysis. However, the majority of documented methods still use artificial precursors, show fluorescence quenching instead of fluorescence enhancement, and have lack validation using real agricultural samples.^15^ Bottom-up techniques like hydrothermal and microwave-assisted methods are frequently used to synthesize CDs, particularly when employing sustainable, environmentally friendly precursors.^16^ Red beetroot (*Beta vulgaris*) juice was used as the carbon source in this study, which used a natural and economical synthesis method.^17^ Because of their distinct optical characteristics, high photostability, and abundant surface functional groups (such as –OH and –COOH), which enable strong interactions with target analytes, carbon dots (CDs) have become a promising sensing material. Compared to conventional recognition elements, CDs offer a straightforward, quick, and affordable platform for fluorescence-based detection.^18^

To overcome these obstacles, we have created a portable, smartphone-assisted turn-on fluorescence sensor for α-CYP detection using carbon dots made from green-synthesized beetroot. Without the use of complex instruments, this environmentally friendly platform achieves a sensitive turn-on response by utilizing the phenomenon of aggregation-induced emission enhancement (AIEE). The rapid on-site analysis of pesticide residue is made possible by the system's optimization for direct application to fruit samples. This study offers a validated tool for the field monitoring of pesticide contamination in food and environmental matrices in addition to showcasing a sustainable approach to sensor fabrication.

## 2 Materials and methods

### 2.1 Study design and material selection

The goal of this project was to create a fluorescence-based, portable, environmentally friendly sensing platform for α-CYP detection in agricultural products. Because of its high pigment and carbohydrate content, abundance, and non-toxicity, beetroot (*Beta vulgaris*) was chosen as the carbon precursor to enable the environmentally friendly synthesis of carbon dots (CDs). Because α-cypermethrin (α-CYP) is widely used in Iraqi agriculture and poses health and environmental risks, it was selected as the target analyte. Due to their widespread cultivation in the study area and their vulnerability to pyrethroid pesticide application, oranges were chosen as the test crop.

### 2.2 Materials and chemicals

Fresh beetroot (*Beta vulgaris*) was purchased from a local supermarket in Sulaymaniyah, Iraq. Deionized water was used as the main solvent in all experimental procedures. The commercial α-CYP formulation (α-CYP, 10%) was supplied by the Karbala Agriculture Department in Iraq, while the analytical standard of α-CYP (purity > 99%) was obtained from Dr Ehrenstorfer GmbH, Germany. For pesticide extraction and calibration investigations, Sigma-Aldrich provided high-performance liquid chromatography (HPLC)-grade solvents, such as acetonitrile, methanol, and water (purity > 99.9%). Other reagents, including phosphoric acid (85%, Merck), sodium hydroxide (99%, B.H.D. Company), hydrochloric acid (36% w/v, B.H.D. Company), Supelclean Primary-Secondary Amine solid-phase extraction (PSA SPE; Bulk Packing form, Merck), and ethanol, were used in their analytical-grade form without additional purification. To guarantee uniformity and reproducibility throughout experiments, buffer solutions and other auxiliary chemicals were made in accordance with accepted laboratory practices.

### 2.3 Instruments

A microwave digestion system (Multiwave GO Plus, Anton Paar, Austria) assisted in the green hydrothermal synthesis of the emissive CDs. This system offered improved energy efficiency, regulated pressure conditions, and increased thermal uniformity. After being used as a natural carbon precursor and heated under microwave irradiation, the fresh beetroot extract produced uniformly distributed, highly fluorescent CDs. A PG Instruments T80 double-beam spectrophotometer (UK) was used to record the UV-visible absorbance spectra of the synthesized CDs and α-CYP, with measurements made between 200 and 800 nm. To assess the pH-dependent behavior of the CDs and their interaction with the pesticide, pH readings were taken using a Hanna Instruments pH meter and a variety of buffer systems. A Shimadzu RF-5301 spectrofluorometer (Japan) with a 450 W xenon arc lamp as the excitation source was used to record photoluminescence (PL) spectra. The slit widths were 5 mm for both the excitation and emission paths. The excitation wavelength range was 360–460 nm, while emission spectra were recorded from 347 and 450 nm onwards. An iPhone 13 Pro Max camera was used to take pictures of the fluorescent solutions under a UV light in order to visually track the fluorescence response. Red (R), green (G), and blue (B) intensity values were extracted from these images using Color Detector software. The colorimetric analysis of the RGB data supported the qualitative and quantitative assessments of fluorescence quenching in response to α-CYP.

### 2.4 Synthesis of CDs using red beetroot *via* the microwave-assisted hydrothermal method

**Fig. 2:**
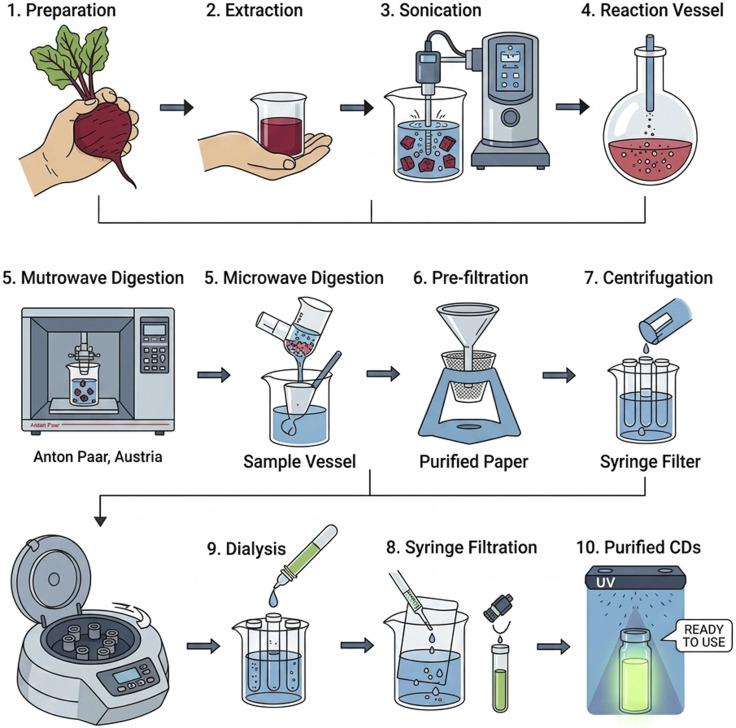
Microwave-assisted hydrothermal synthesis of CDs from red beetroot.

Fresh red beetroot (*Beta vulgaris*) was used as a sustainable carbon precursor in a quick and energy-efficient microwave digestion system for the first time to create CDs. 10.0 g of finely chopped beetroot was first homogenized with 70 mL of deionized water under vigorous stirring for 20 minutes and then ultrasonically agitated for 1 hour. The resulting mixture was transferred to TFM digestion vessels and subjected to microwave-assisted hydrothermal carbonization in a Multi-wave GO Plus microwave digestion system (Anton Paar, Austria). The temperature was increased within 15 min from starting, and it ramped to 180–200 °C over 20 min and maintained for 10 min under controlled pressure. The solution was cooled, centrifuged (10 000 rpm, 30 min), filtered (0.22 µm), and dialyzed (3.5 kDa membrane, 72 h), as illustrated in Fig. 2. The purified CDs were stored at 4 °C until use.

#### 2.4.1 Proposed chemical transformation during carbonization

C_6_H_12_O_6_ represents the carbohydrate-rich compounds in beetroot undergoing dehydration, aromatization, and surface passivation with the functional groups (–OH, –COOH, and –NH_2_) derived from beetroot proteins and amino acids, as shown in eqn (1).1
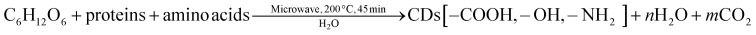


### 2.5 Experimental variables

• **Independent variables:**

∘ α-CYP concentration (0–80 µmol L^−1^)

∘ pH (3–9)

∘ Reaction time (0–10 min)

∘ Pesticide type (for selectivity tests).

• **Dependent variables:**

∘ Photoluminescence (PL) intensity at 450 nm

∘ RGB intensity values (R + G) from smartphone images.

• **Controlled variables:**

∘ UV excitation wavelength (365 nm)

∘ Synthesis temperature and time

∘ Sample extraction volume and method

∘ Instrument calibration and environmental lighting during imaging.

### 2.6 Fluorescence sensing of α-CYP using CDs

Monitoring the impact of the pesticide on the photoluminescence (PL) intensity of the synthesized CDs at room temperature allowed for the fluorescence-based detection of α-CYP. In an experiment, 1 mL of phosphate-buffered saline (PBS, 10 mM, pH 7.2) was mixed with 10 µL of a CD solution (0.3 mg mL^−1^). To create working solutions at different concentrations ranging from 0 to 80 µmol L^−1^, α-CYP was dissolved in a 1 : 1 mixture of deionized water and acetonitrile. Following the addition of the pesticide to the CD solution, the mixture was allowed to sit at room temperature for 3 minutes. A spectrofluorometer was then used to record the fluorescence spectra with an excitation wavelength of 347 nm and an emission scan from 360 to 700 nm. In order to assess the hydrophobic interaction and aggregation restriction effect caused by α-CYP, the CDs showed a clear emission peak at 450 nm.

### 2.7 Selected pesticide

α-CYP was chosen as the target analyte for this investigation because of its extensive agricultural application and environmental significance. Alpha-Cyper 10% EC, a commercial formulation, was utilized and applied at the suggested rate of 150 mL per 100 liters of water. In compliance with the directives from the Kurdistan Regional Government's (KRG) Ministry of Agriculture, the pesticide was acquired from a local vendor in Erbil, Iraq.

### 2.8 Field experiments and sample collection

A private citrus orchard in Kalar, Sulaymaniyah, Iraq, served as the site of the field tests. The Garmian summer orange tree in the orchard was 12–13 years old and stood between 1.4 and 1.6 meters tall. In addition to untreated control trees, the experimental design used a randomized block layout with three replicates, each of which contained three trees per treatment. Using a backpack hand sprayer with a single nozzle, pesticide applications were carried out in accordance with the Ministry of Agriculture and Water Resources' recommendations. During the fruiting period, the trees received a single spraying. Representative orange samples were randomly selected from each treatment plot at various intervals following pesticide application, including 2 hours, 1 week, 2 weeks, and 3 weeks. CDs made from beetroot juice were used in a fluorescence-based method for detecting pesticide residues. Green synthesis was used to prepare the CDs, and a smartphone camera was used to detect changes in fluorescence intensity in a UV light cabinet. This method allowed for the direct monitoring of pesticide residues from the treated fruit extracts and offered a straightforward, economical, and ecologically friendly substitute for traditional chromatographic techniques.

### 2.9 Extraction and residue analysis

A modified QuEChERS method,^19^ was used to process orange samples. Briefly, 15 mL of acetonitrile acidified with 1% acetic acid was added to a 50 mL centrifuge tube containing 10.0 g of the homogenized fruit. For a minimum of 1 minute, the mixture was shaken vigorously. 1.0 g of sodium acetate and 4.0 g of MgSO_4_ were then added. The mixture was centrifuged for 5 minutes at 3000 rpm following 5 minutes of vigorous shaking. For additional cleanup, a 5 mL portion of the supernatant was transferred to a 15 mL tube with 50 mg of PSA, 10 mg of graphitized carbon black, and 150 mg of anhydrous magnesium sulfate. The mixure was subjected to continuous agitation for ten minutes to ensure complete extraction of the analyes into the solvent phase, this mixture was centrifuged once at 6000 rpm for 10.0 min the liquid solution was collected for analysis.

**Fig. 3:**
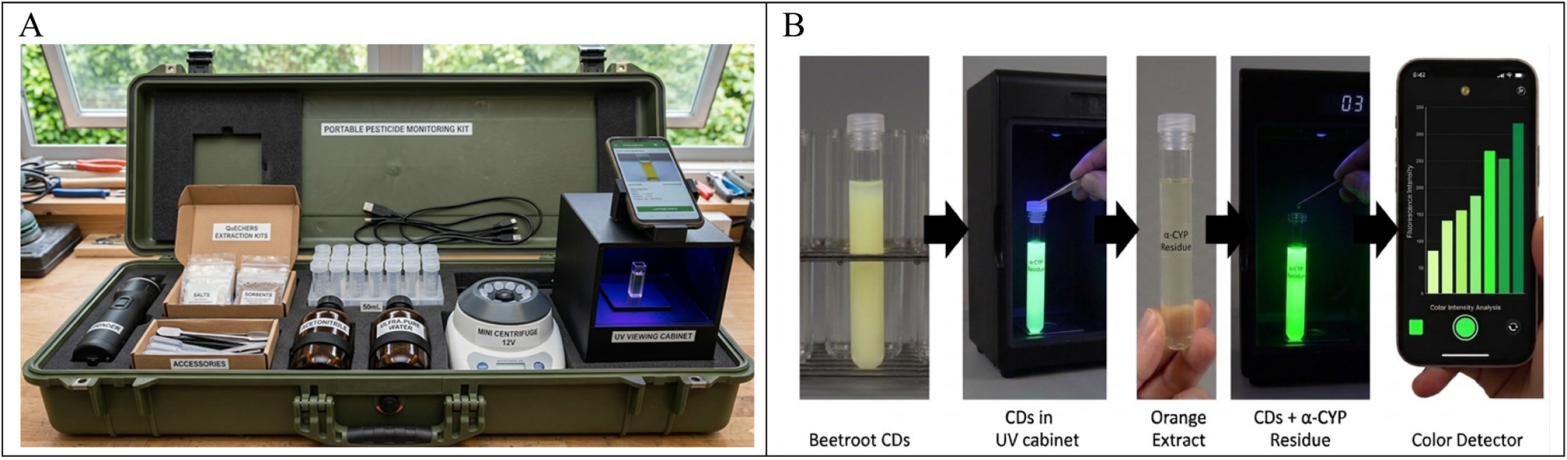
(A) Portable pesticide monitoring box suggested for future and (B) portable detection of the CDs' fluorescence.

The system is housed in a compact, durable carrying case (0.6 m *L* × 0.25 m *W* × 0.25 m *H*) equipped with ergonomic handles to facilitate easy transport for field-based analysis (see Fig. 3A). It held all the important parts of the kit, such as a rechargeable-battery-powered mini sample grinder, a centrifuge, and a compact UV illumination chamber. This setup, along with RGB analysis on a smartphone, allowed us to prepare and detect samples on the spot. This shows how the suggested method can be used to make portable analytical systems that can test a wide range of pesticides in the future. They would allow quick, on-site testing and help improve safety management for both agriculture and the environment.

### 2.10 Statistical analysis

Origin Ro 2021 was used for all data analysis and graphical plotting. The mean, standard deviation (SD), relative standard deviation (RSD), standard error (SE), limit of detection (LOD), and limit of quantitation (LOQ) were among the statistical parameters that were computed. To evaluate the analytical performance of the carbon dot-based sensing system, calibration curves and fluorescence quenching plots were created using Origin Pro-integrated linear regression tools.

### 2.11 Smartphone-based fluorescence detection

The fluorescence response of the CDs was observed under a UV lamp (365 nm) and recorded using a high-resolution iPhone 13 Pro Max camera without flash to avoid interference, as shown in Fig. 3B, allowing for real-time and portable detection. An RGB-based color intensity evaluation app (Color Detector) was used for image analysis, enabling the estimation of the pesticide concentration based on a drop in the fluorescence intensity.

## 3 Results and discussion

The results show that a low-cost, sensitive, and environmentally friendly fluorescent sensor for α-CYP detection has been successfully developed. The CDs made from beetroot exhibit good optical qualities, a high quantum yield, and a strong aggregation-induced emission enhancement (AIEE) interaction with α-CYP. The method is perfect for field applications in low-resource environments because it uses a smartphone camera under a UV light to enable portable, on-site detection while also avoiding the need for sophisticated equipment like GC-MS or HPLC.

### 3.1 Characterization of CDs

**Fig. 4:**
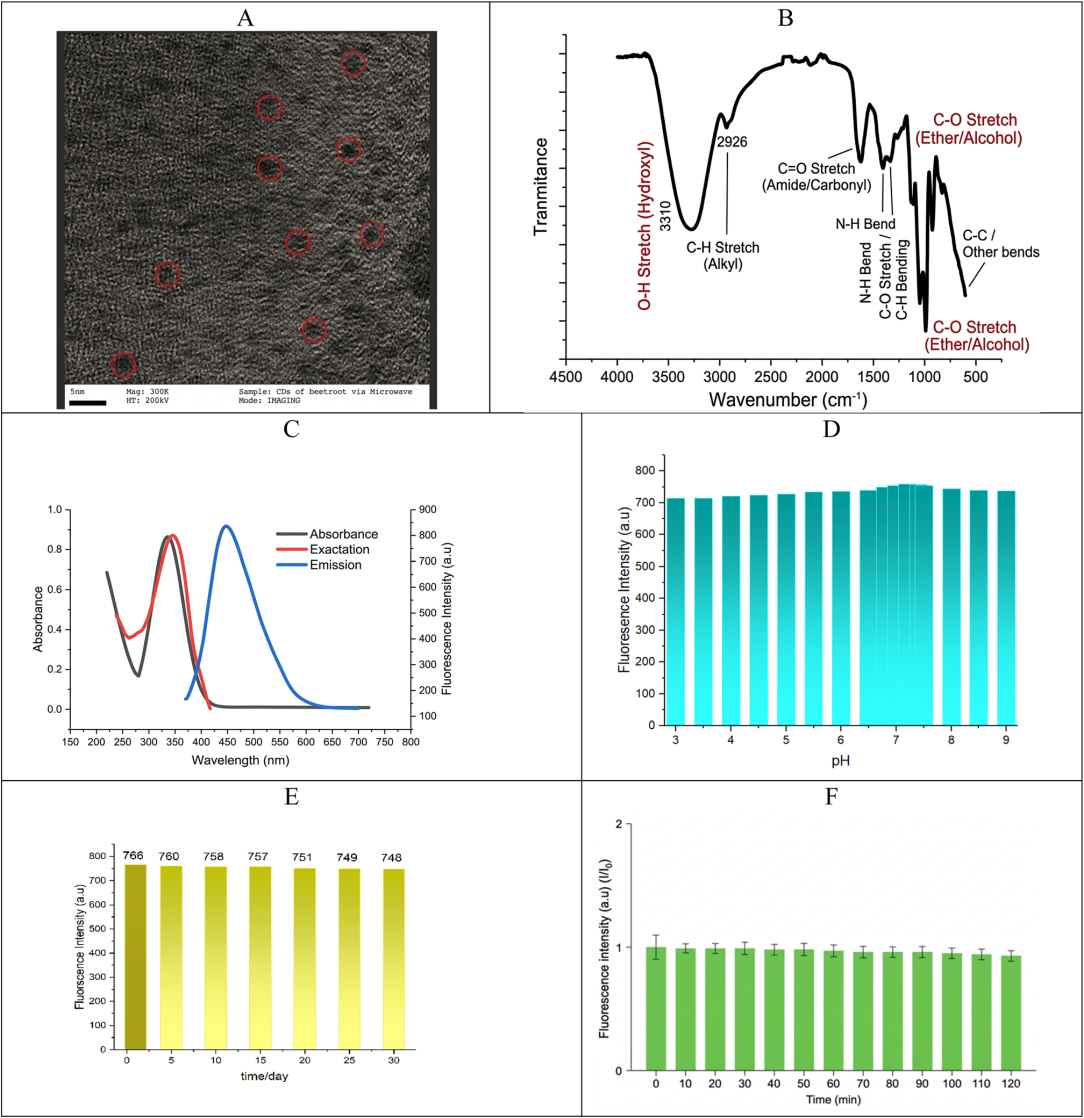
(A) HR-TEM image of the CDs synthesized by the microwave-assisted hydrothermal method; the scale bar is 5 nm. (B) FTIR spectrum of the CDs synthesized from red beetroot. (C) UV-vis, excitation and fluorescence spectra of CDs. (D) Effect of pH on the photoluminescence (PL) intensity of the synthesized CDs. (E) Fluorescence intensity after one-month storage under ambient conditions. (F) Photostability tests under continuous UV exposure.

Numerous characterization techniques were used to verify the successful synthesis and characteristics of CDs. Based on the previously discussed characterization results, the synthesized carbon dots (CDs) exhibit favorable optical properties suitable for pesticide sensing. As seen in Fig. 4A, the HR-TEM image of the carbon quantum dots (CQDs) exhibits a generally spherical morphology with good dispersion. The diameters of the marked CQDs are estimated to be between 3 and 7 nm, using the scale bar (5 nm) as a reference. The particles' uniform size and shape point to a regulated synthesis procedure carried out under hydrothermal conditions with the aid of microwaves. The green fluorescence behavior of quantum dots under UV light is supported by this nanoscale size range.

A broad O–H/N–H stretching band at about 3400 cm^−1^ can be seen in the synthesized carbon quantum dots' FTIR spectrum (Fig. 4B), which indicates the presence of hydroxyl and amine groups. C–H stretching vibrations are the cause of the tiny peak close to 2900 cm^−1^. The carbonyl group's C=O stretching is represented by the peak at 1700 cm^−1^, whereas C=C stretching is associated with the band at 1600 cm^−1^. The presence of C–N and C–O bonds is confirmed by the peaks between 1400 and 1000 cm^−1^. These findings demonstrate the CDs' rich surface functionalization, which enhances their strong fluorescence and good dispersibility. Consequently, the FT-IR spectrum demonstrates that the CDs' surfaces contain carboxyl, carbonyl, and hydroxyl groups, which affords them excellent water solubility and stability in environmental solutions.

The CDs' optical characteristics, such as stability, emission spectra, and UV-vis absorption, were examined. The UV-vis spectrum of CDs shows two characteristic absorption peaks at approximately 222 and 336 nm, which are related to the π–π* transition of aromatic C=C bonds and the n–π* transition of C=O bonds, respectively, as shown in Fig. 4C. The synthesized CDs exhibit strong fluorescence emission peaks that are characterized of high-quality carbon-based fluorophores. Additionally, Fig. 4C demonstrates that the ideal excitation and emission spectra of synthesized CDs are measured at wavelengths of 347 nm and 444 nm, respectively.

Furthermore, the impact of the pH and time on the emission intensity of the synthesized CDs was measured in order to discuss the stability of fluorescent CDs. The emission intensity of CDs increases as the pH values rise from 3 to 9.0, as shown in Fig. 4D. The CD emission intensity then drops as the pH increases to levels greater than 7.2. A pH of 7.2 is found to be the ideal value for achieving the high emission intensity of CDs. Due to different functional groups found in the precursor's proteins and carbohydrates, the majority of natural CDs self-passivate. This demonstrates that the produced CDs may be appropriate for cellular and bioimaging applications and stable under most physiological circumstances.

CDs' excellent photostability is confirmed by their photoluminescence (PL) spectra at an excitation wavelength of 348 nm, which show no discernible change in the emission intensity after a month of storage under ambient conditions (Fig. 4E). The slight decrease in the fluorescence intensity may be attributed to minor surface photo-oxidation under continuous UV exposure; however, the CDs maintain high structural and optical stability (Fig. 4F).

Using quinine sulfate (QS) as a reference standard, the quantum yield (*Φ*) of the CDs was calculated (*Φ*_ref_ = 0.546 in 0.1 M H_2_SO_4_). Eqn (2) was used to determine the relative quantum yield:2
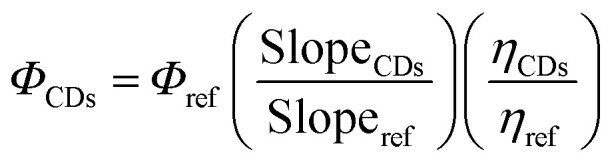


**Fig. 5:**
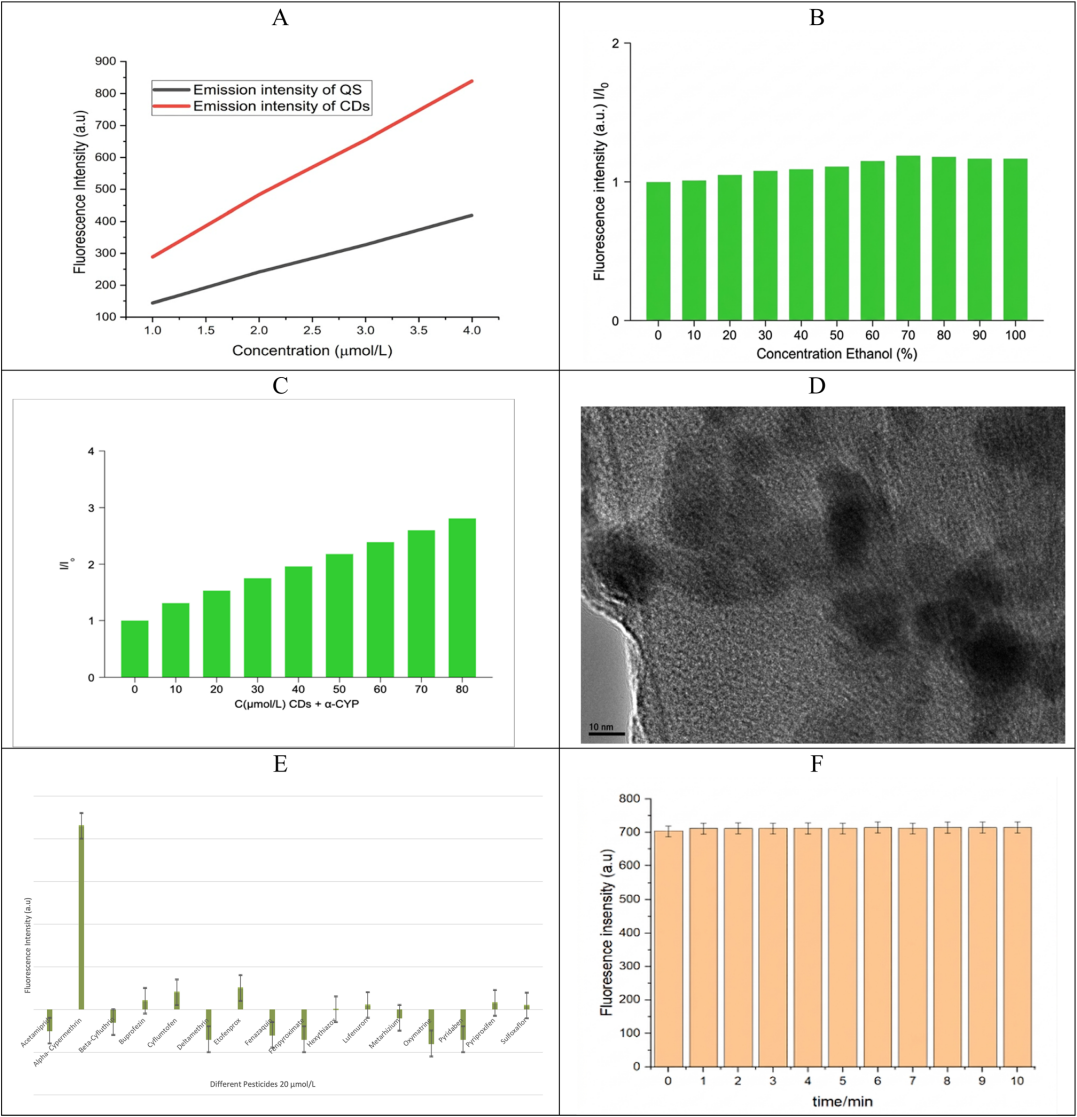
(A) Fluorescence intensity of the CDs and quinine sulfate (QS) for the determination of the quantum yield, (B) solvent-dependent fluorescence behavior, (C) concentration-dependent fluorescence studies showing the formation of emissive aggregates, (D) TEM image showing the CDs after interaction with α-CYP, (E) investigation of the selectivity of different pesticides, and (F) reaction time between the CDs and α-CYP.

The experimental values (slope_CDs_ = 182.2, slope_ref_ = 495; refractive index: *η*_CDs_ 1.61 and *η*_ref_ = 1.4) (Fig. 5A), the quantum yield of the CDs is determined to be *Φ*_CDs_ = 26.6%. This moderate quantum yield suggests efficient radiative recombination, likely influenced by surface functional groups and defect states. The refractive index correction accounts for solvent differences, and the use of QS as a reference ensures comparability with standard fluorophores.

Our QY is competitive and, in many cases, better than that of other carbon dots made from natural sources for sensing. For example, carbon dots made from rose petals have a QY of 9.6%,^20^ and those made from lemon peels have a QY of 16.9%.^21^ Our CD's QY is also much higher than that of CDs made from date seeds (5.16%, ref. 22) and coffee grounds (6.01%, ref. 23).

### 3.2 Aggregation-induced emission enhancement (AIEE) mechanism

The fluorescence enhancement observed in the presence of α-cypermethrin is attributed to an aggregation-induced emission enhancement (AIEE) mechanism. In the dispersed state, there are abundant surface functional groups, including hydroxyl (–OH), carboxyl (–COOH), and carbonyl (C=O) groups. These functional groups act as active sites for interaction with α-cypermethrin through hydrogen bonding and π–π interactions. Such interactions facilitate the aggregation of CDs and restrict intramolecular motion, leading to enhanced fluorescence emission.

This hypothesis were carried out in order to confirm it. Initially, different polarity solvent systems were used to study the fluorescence behavior of CDs. A notable increase in the fluorescence intensity, reaching a maximum at 70%, is seen as the fraction of poor solvent rises (Fig. 5B). Additionally, concentration-dependent fluorescence studies show that the formation of emissive aggregates is supported by an increase in the emission intensity with increasing CD concentration (Fig. 5C). Furthermore, TEM images show that the CDs are evenly distributed when α-cypermethrin is not present, but after the interaction with α-CYP, clear aggregates are seen (Fig. 5D).

All of these findings support the idea that the AIEE mechanism, in which aggregation limits nonradiative pathways and promotes radiative recombination, is the source of the fluorescence enhancement.

### 3.3 Selectivity and analytical efficiency of the CD probe for α-CYP sensing

A number of widely used pesticides in Iraq were examined as possible interferents in order to examine the selectivity of the suggested sensing system. Acetamiprid, α-CYP, beta-cyfluthrin, buprofezin, cyflumetofen, deltamethrin, etofenprox, fenazaquin, fenpyroximate, hexythiazox, lufenuron, *Metarhizium*, oxymatrine, pyridaben, pyriproxyfen, and sulfoxaflor were among them. Similar to α-CYP, each interfering pesticide was prepared at a concentration of 20 µmol L^−1^ and added to the CDs. The prepared CDs show a notable increase in the fluorescence intensity (turn-on fluorescence) when α-CYP is gradually added. Although deltamethrin exhibits structural similarities with cypermethrin, due to its bulkier structure and higher hydrophobicity, it exhibits weaker interaction with the CD surface and does not effectively induce aggregation. As shown in Fig. 5E, the presence of α-CYP leads to a significant increase in fluorescence intensity (turn-on effect), whereas other pesticides showed negligible interference.

The reaction time of CDs with α-CYP was examined and optimized in order to determine the ideal conditions for the CD-based detection of α-CYP. When a solution containing 10 µmol per L α-CYP was added, the CDs' emission intensity instantly increased, as shown in Fig. 5F. As a result, 3 minutes were chosen as the ideal CD reaction time with α-CYP for the subsequent research. Additionally, the optimal conditions were used for the quantitative measurement of α-CYP.

The proposed CDs' selectivity was tested against a number of interferents. It shows high specificity for α-CYP. The recovery studies show values between 92.3% and 98.2% with a low RSD, which shows that the results are accurate and precise, as shown in Table 3.

To find out what the matrix effect was, we compared calibration curves in a pure solvent and real sample matrices. A very small difference in the slope is seen, which means that there is very little matrix interference and that the method is reliable for analyzing real samples.

### 3.4 Fluorescence response to α-CYP

**Fig. 6:**
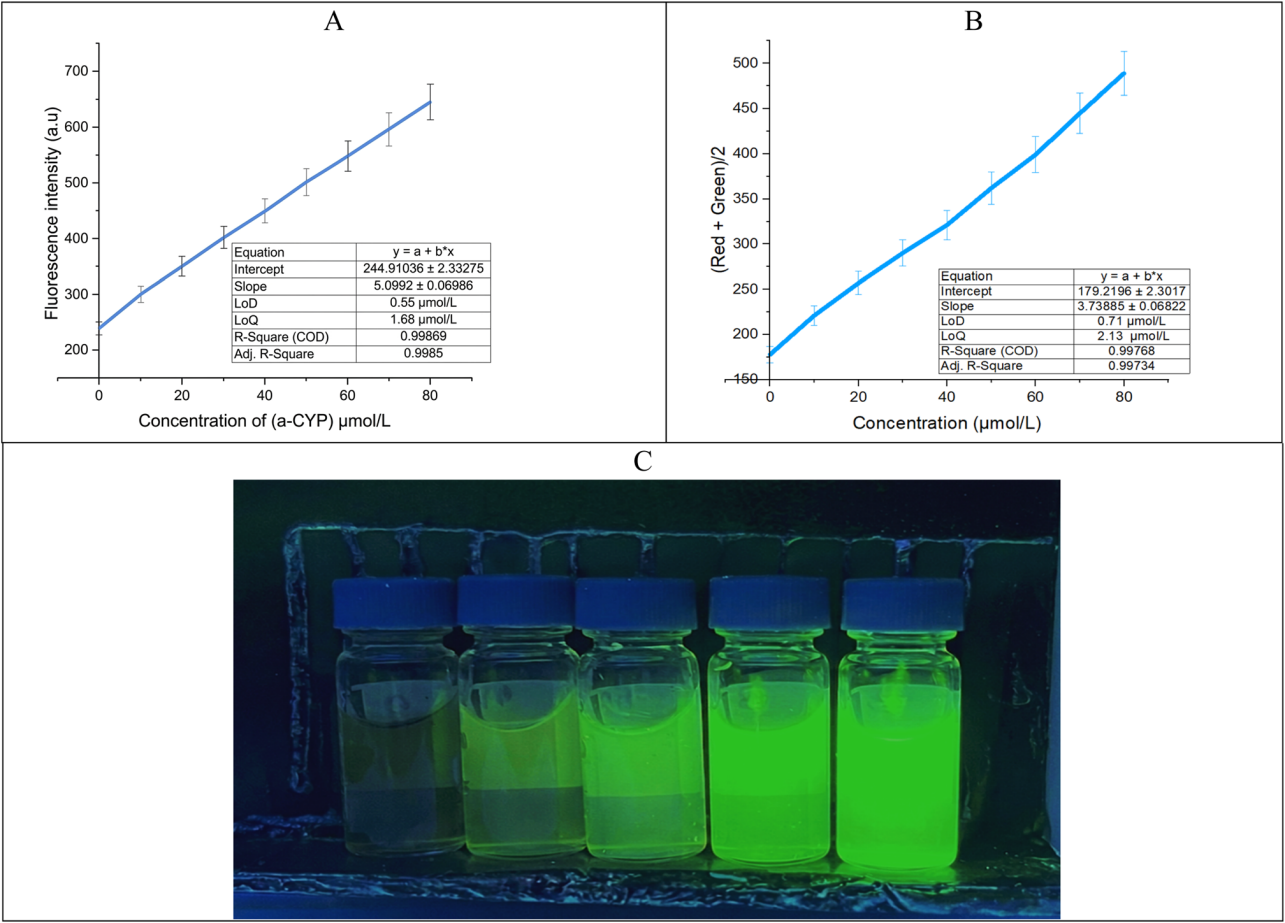
(A) CDs with concentrations of α-CYP between 0 and 80 µmol L^−1^, (B) calibration curve, the average of the red and green channels (R + G) was calculated and plotted against varying concentrations of (α-CYP) (0–80 µmol L^−1^), and (C) the “turn-on” fluorescence intensity of the CDs under UV light with α-CYP.

With the addition of α-CYP, the PL intensity of the beetroot-derived carbon dots (CDs) exhibited a concentration-dependent response, attributed to aggregation-induced emission enhancement (AIEE) and electron transfer interactions between α-CYP and surface functional groups of the CDs. The PL spectra obtained for α-CYP concentrations ranging from 0 to 80 µmol L^−1^ (Fig. 6A) revealed a systematic variation in the emission intensity with increasing pesticide concentration. The corresponding calibration curve displayed excellent linearity (*R*^2^ = 0.9978) over this range, with a limit of detection (LOD) of 0.55 µmol L^−1^ and a limit of quantification (LOQ) of 1.80 µmol L^−1^. Notably, both values are lower than the European Union's maximum residue limit (MRL) for α-CYP in citrus fruits (10 µmol L^−1^),^24,25^ underscoring the suitability of the proposed method for regulatory compliance monitoring.

### 3.5 Performance comparison with existing methods

The developed sensor is found to be comparable to other analytical tools (Table 1), particularly in terms of its portability and low cost. This method's LOD and LOQ values are not as high as those of a laboratory-bound HPLC or GC system. However, they are still within the range that is good within the range deemed acceptable. The fact that it is less sensitive than some of the standard methods is a trade-off against its usefulness and ability to analyze data in real time. Hence this technology is a big step toward making easy-to-use and quick analytical tools for environmental monitoring and food safety.

**Table 1:** Comparison of the LOD and LOQ of the proposed method and previously reported techniques

Technique	Sample type	LOD (µmol L^−1^)	LOQ (µmol L^−1^)	Reference
HPLC	Water	0.113	0.339	25
HPLC	Soil	0.05	0.150	26
GC analysis	Bullfrog organ	0.00480	0.0144	27
GC analysis	Vegetables	0.0120	0.036	28
GC analysis	Vegetables	0.0240	0.0720	29
GC with electron capture detection (GC-µECD)	AL mosquito nets	22.58	67.74	30
Photochemically induced fluorescence (PIF)	Natural water	0.325	0.767	31
Thioglycolic acid-caped Mn-doped ZnS quantum dots (TGA@Mn–ZnS-QDs)	Tomato, okra, pea, spinach, soil, and water	0.317	1.046	32
Optical polymer sensor based on MIP-UiO66/Fe_3_O_4_ for the detection of cypermethrin	Agricultural products	0.576	1.900	33
Spectrofluorometer sensor (CDs from beetroot)	Orange	0.55	1.68	Present study
Smartphone-based RGB sensor (CDs from beetroot)	Orange	0.71	2.13	Present study

### 3.6 Real sample analysis

We took a baseline measurement before preparing and analyzing the samples for the dissipation study. We made a blank extract from an untreated orange using the same QuEChERS method and then used the smartphone-based method to study it, along with the spiked and field samples. This analysis demonstrated that the blank orange matrix did not display significant fluorescence under UV excitation conditions, nor did it induce any substantial interference with the specific fluorescence response of the carbon dots to α-CYP.

A field test was carried out using orange fruits sprayed with α-CYP, in compliance with the official Iraqi Ministry of Agriculture's pesticide application regulations, in order to assess the developed sensing system's practical applicability. In a citrus orchard, the pesticide was sprayed using standard methods at the recommended dosage. Representative fruit samples were taken for residue analysis at predetermined intervals of 0 (initial), 1, 2, and 3 weeks after spraying (Table 2). As previously mentioned, a modified QuEChERS method^19^ was used to extract pesticide residues from orange samples. The synthesized CDs derived from beetroot were then used to analyze the resulting extracts under a UV light. A smartphone-based detection setup was used to record the RBG color, and a fluorometer instrument was used to record the fluorescence emission. The pre-established calibration curve was used to correlate changes in the emission intensity with the pesticide concentration.

3 *y* = 5.0992*x* + 244.9103

**Table 2:** Measured residue levels of α-CYP in orange samples over time

Intervals (weeks)	Residues (µmol L^−1^)	Loss%	Persistence%
Initial	4.93	1.4	98.6
1	3.01	39.8	60.2
2	0.98	80.4	19.6
3	0	100.0	0

Based on the calibration curve slope and the standard deviation of the blank, the LOD is 0.55 µmol L^−1^, and the LOQ is 1.68 µmol L^−1^, as shown in eqn (3), indicating an acceptable amount compared to previously reported techniques for detecting α-CYP, as reported in Table 2. These results show that the constructed probe has sufficient selectivity and sensitivity for α-CYP detection.

In the third week after application, the residue concentration gradually decreases to undetectable levels, according to the data. This pattern, which corresponds with established environmental behavior and safety intervals, validates the pesticide's gradual degradation and/or dissipation.

Using spiked orange samples at four distinct concentrations, a recovery study was conducted to confirm the extraction effectiveness and dependability of the sensing technique. Table 3 provides a summary of the findings:

**Table 3:** Spiked recovery of α-CYP using the naturally synthesized CDs

Matrix	Spiked (µmol L^−1^)	Found (µmol L^−1^)	Recovery (%)	RSD (%)
Orange	5	4.91	98.2	1.04
Orange	10	9.50	95.0	3.04
Orange	20	18.8	94.0	2.72
Orange	30	27.7	92.3	2.67

The high recovery values (above 90%) and low relative standard deviations (RSD < 4%) demonstrate that the fluorescence-based sensing system is both accurate and reproducible. These findings support the suitability of this eco-friendly, smartphone-assisted detection approach for the on-site monitoring of pesticide residues in real agricultural produce.

### 3.7 Smartphone-based detection and RGB color analysis

A colorimetric detection method that works on smartphones was created as a quick, cheap, and portable way to check for α-CYP residues on-site instead of using a spectrofluorometer in a laboratory. This method uses the aggregation-induced emission enhancement (AIEE) effect of carbon dots (CDs) made from beetroots. When α-CYP is added, the “turn-on” fluorescence intensity of the CDs under UV light increases (Fig. 6C), which is easy to see with the naked eye (Fig. 3B).

A detailed and standardized protocol was created for the smartphone imaging setup to make sure that the measurements could be repeated. All pictures were taken with an iPhone 13 Pro Max in controlled, standard ambient lighting to avoid interference from outside light. To keep the smartphone camera and the sample at a constant distance and angle, a custom-made, 3D-printed stand was used. This avoided changes in image capture. The camera settings were locked, the flash was turned off, and the focus and white balance were manually set so that all measurements would be the same.

We plotted a calibration curve by plotting the average *I*_R+G_ against different α-CYP concentrations (0–80 µmol L^−1^) (Fig. 6B). The regression shows a strong linear relationship, which proves that RGB analysis can be used to semi-quantitatively detect α-CYP.

It is important to note that the smartphone-based method gives slightly higher LOD/LOQ values (0.71/2.13 µmol L^−1^) than the spectrofluorometer (0.55/1.74 µmol L^−1^). The optical system of the spectrofluorometer is built to be more sensitive and accurate than the camera on a consumer-grade smartphone. The smartphone-assisted method is not as sensitive, but it is a useful and effective tool for quickly screening for residues on-site in field applications where real-time decisions are important. The successful combination of fluorescence sensing and computer vision-based analysis shows that smartphone-assisted tools can be used more widely for monitoring food and environmental safety.

## 4 Conclusion

This study effectively illustrated the creation of a green, fluorescence-based sensor that uses CDs made from beetroot juice to detect α-CYP. Highly fluorescent, yellow-emissive CDs with superior stability and dispersibility were produced using both hydrothermal and microwave-assisted techniques. Because of hydrophobic interaction mechanisms, the prepared CDs showed increased fluorescence in the presence of α-CYP, enabling the development of a sensitive and dependable detection system. Optimization, selectivity, and real-sample analysis were used to thoroughly validate the sensing platform. In orange fruit matrices, the technique demonstrated outstanding recovery and reproducibility, which is consistent with real-world farming circumstances. Additionally, semi-quantitative detection was made possible by the combination of a smartphone camera and RGB analysis software, offering an approach that is accessible and easy to use for in-field applications. By fusing green synthesis, smartphone-based detection, and real-world validation, this work advances sustainable sensor technology. For tracking pesticide residues in food and environmental samples, the suggested approach presents a viable substitute for traditional instrumentation.

## Conflicts of interest

There are no conflicts to declare.

## Data Availability

All data generated and analyzed during this study have been fully included within the main manuscript. All results, including experimental data and analyses, are presented in the article itself.
